# Solving robotics tasks with prior demonstration via exploration-efficient deep reinforcement learning

**DOI:** 10.3389/frobt.2025.1682200

**Published:** 2026-01-12

**Authors:** Chengyandan Shen, Christoffer Sloth

**Affiliations:** 1 Unicontrol ApS, Odense, Denmark; 2 SDU Robotics, Maersk McKinney Møller Institute, University of Southern Denmark, Odense, Denmark

**Keywords:** deep reinforcement learning, learning from demonstration, automation in construction, robotics, sim-to-real

## Abstract

This paper proposes an exploration-efficient deep reinforcement learning with reference (DRLR) policy framework for learning robotics tasks incorporating demonstrations. The DRLR framework is developed based on an imitation bootstrapped reinforcement learning (IBRL) algorithm. Here, we propose to improve IBRL by modifying the action selection module. The proposed action selection module provides a calibrated Q-value, which mitigates the bootstrapping error that otherwise leads to inefficient exploration. Furthermore, to prevent the reinforcement learning (RL) policy from converging to a sub-optimal policy, soft actor–critic (SAC) is used as the RL policy instead of twin delayed DDPG (TD3). The effectiveness of our method in mitigating the bootstrapping error and preventing overfitting is empirically validated by learning two robotics tasks: bucket loading and open drawer, which require extensive interactions with the environment. Simulation results also demonstrate the robustness of the DRLR framework across tasks with both low and high state–action dimensions and varying demonstration qualities. To evaluate the developed framework on a real-world industrial robotics task, the bucket loading task is deployed on a real wheel loader. The sim-to-real results validate the successful deployment of the DRLR framework.

## 1 Introduction

Model-free deep reinforcement learning (DRL) has shown great potential in learning continuous control tasks in robotics (Allshire et al., 2022; Qi et al., 2023; Rudin et al., 2022; Haarnoja et al., 2018a; Nguyen and La, 2019; Ibarz et al., 2021). However, there remain challenges that limit the widespread applicability of these methods in real-world robotic applications. One major challenge is the poor sample efficiency of learning with model-free DRL; even relatively simple tasks can require millions of interaction steps, while learning policies from high-dimensional observations or complex environments may require significantly more interactions (Haarnoja et al., 2018b; Osinski et al., 2020; Raffin et al., 2021). A primary cause for the poor sample efficiency is on-policy learning (Haarnoja et al., 2018b) since some of the most widely used DRL algorithms, such as A3C (Mnih et al., 2016) and PPO (Schulman et al., 2017), require new interactions with the environments for each gradient step. Consequently, on-policy DRL is often impractical for real-world systems as allowing untrained policies to interact with real systems can be both costly and dangerous. Even when learning occurs solely in simulation, it is still preferred to utilize previously collected data instead of starting from scratch (Levine et al., 2020). On the other hand, off-policy DRL methods improve sample efficiency by reusing past experience and have demonstrated strong performance on continuous control tasks (Lillicrap et al., 2015; Fujimoto et al., 2018; Haarnoja et al., 2018b; Fujimoto et al. 2019; Fujimoto and Gu, 2021; Kumar et al., 2020). However, for complex robotics tasks where data collection itself is expensive, e.g., in construction machines, educational agents, or medical devices, even off-policy approaches become costly when the DRL policy requires extensive explorations. Under these scenarios, improving exploration efficiency is as crucial as sample efficiency to reduce the exploration needed for achieving a good policy.

Therefore, effectively leveraging prior demonstrations to facilitate efficient exploration is considered a promising strategy for the broad application of off-policy DRL in real-world industrial robotics. Two main research directions have emerged to achieve this goal:

### 1.1 Offline-to-online DRL

Pretraining DRL with prior expert demonstrations, along with continuous training with online data, has shown its impressive performance in exploration efficiency (Vecerik et al., 2017; Nair et al., 2020; Uchendu et al., 2023; Zhou et al., 2024; Goecks et al., 2019; Lee et al., 2022). Early studies initialize training by mixing offline demonstrations and online interaction in the replay buffer and use a prioritized replay mechanism to enable the reinforcement learning (RL) policy for efficient exploration (Vecerik et al., 2017; Song et al., 2022). More recent approaches separate offline pretraining from online fine-tuning and report superior exploration efficiency (Gao et al., 2018; Goecks et al., 2019; Nair et al., 2020; Lee et al., 2022). In offline training, a behavior cloning (BC) loss or Kullback–Leibler (KL) divergence is typically used to encourage the RL policy to closely follow the behavior policy, which is used to generate the demonstrations, thereby facilitating efficient exploration in online interactions. However, when transferring to the online interacting phase, some methods are required to “recalibrate” the offline Q-estimates to the new online distribution to maintain learning stability and mitigate forgetting of pre-trained initializations (Nair et al., 2020; Uchendu et al., 2023; Ball et al., 2023).

### 1.2 DRL-Ref policy

Some novel studies have proposed to explicitly integrate a reference policy, trained from the prior demonstration to guide DRL training (Zhang et al., 2023; Hu et al., 2024). In these works, a stand-alone reference policy is trained using offline demonstration and then used to provide additional guidance in the DRL online learning phase. In this work, we consider the imitation bootstrapped reinforcement learning (IBRL) framework as an ideal approach for learning robotics tasks with prior demonstrations as it prevents catastrophic forgetting of pre-trained initializations and automatically balances offline and online training (Hu et al., 2024).

**FIGURE 1:**
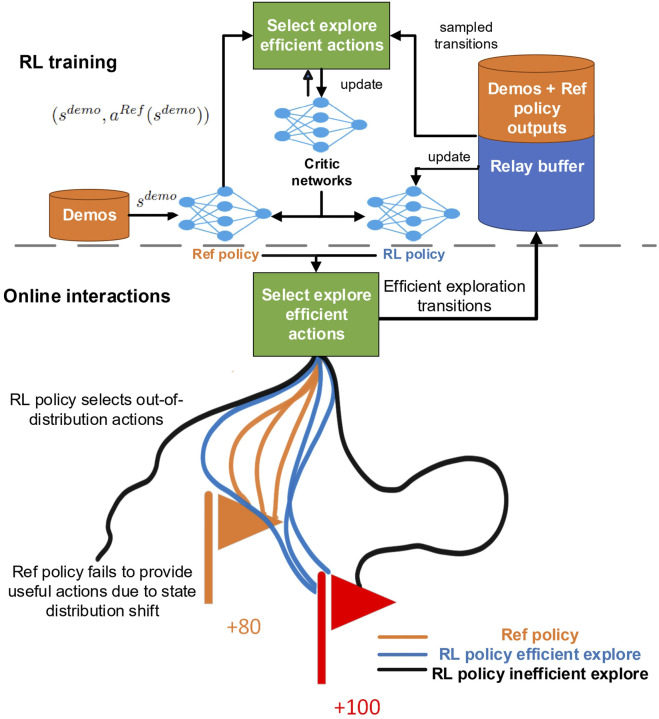
Overview of the proposed exploration-efficient DRLR framework. The proposed framework extends a sample-efficient DRL-Ref method with a simple action selection module to mitigate inefficient explorations caused by (1) bootstrapping errors leading to the RL policy selecting out-of-distribution actions; (2) Ref policy failing to provide good actions under state distribution shifts.

However, the IBRL framework is built on off-policy RL and imitation learning (IL). It risks the same challenges posed by bootstrapping errors in off-policy RL (Kumar et al., 2019, Kumar et al., 2020, Fujimoto et al., 2019, Fujimoto and Gu, 2021), where the target critic and actor networks are updated using out-of-distribution (OOD) actions with overestimated Q-values (Kumar et al., 2019, Kumar et al., 2020). Meanwhile, the IL policy in IBRL could also face the state distribution shift (Hussein et al., 2017), when OOD actions keep getting selected. To address these challenges, in this work, we propose an exploration-efficient DRL with reference (DRLR) policy framework, as shown in Figure 1, and summarize our contributions as follows:

1. Identify and analyze the main cause of the failure cases trained with the IBRL framework: distribution shift due to the bootstrapping error.
2. Propose a simple action selection module and use a maximum entropy RL to mitigate inefficient explorations caused by bootstrapping errors and convergence on a sub-optimal policy due to overfitting.
3. Demonstrate the effectiveness and robustness of the proposed framework on tasks with both low and high state–action dimensions and demonstrations of different quality.
4. Showcase an implementation and deployment of the proposed framework on a real industrial task.

## 2 Problem statement

**FIGURE 2:**
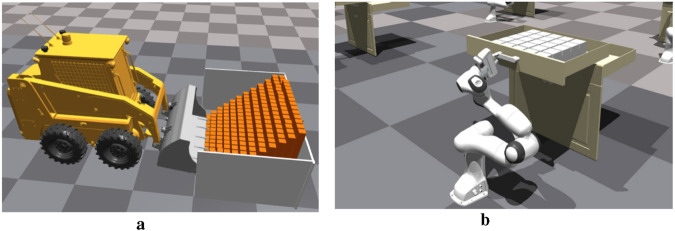
Selected tasks for testing the proposed framework. **(a)** Bucket loading. **(b)** Open drawer.

The proposed framework is generalized toward learning robotics tasks with the following problems: 1) collecting a large amount of data is costly. 2) Learning requires extensive interactions. 3) A small number of expert demonstrations are available. Based on the characteristics, bucket loading (Shen and Sloth, 2024a) and open drawer (Makoviychuk et al., 2021a) tasks are selected to evaluate the effectiveness of the proposed framework. The task environments are shown in Figure 2.

Compared to the selected DRL-Ref framework, IBRL, the proposed framework attempts to mitigate distribution shift caused by bootstrapping errors and prevent convergence to a sub-optimal policy from overfitting to the demonstrations.

Bootstrapping error can arise in off-policy RL when the value function is updated using Bellman backups. It occurs because the target value function and policy are updated using OOD actions with overestimated Q-values (Kumar et al., 2010). Studies have shown that bootstrapping error can lead to unstable training and even divergence from the optimal policy (Kumar et al., 2019, Kumar et al., 2020), particularly when the current policy output is far from the behavior policy, which is used to generate the transitions in the replay buffer (Fujimoto et al., 2019; Fujimoto and Gu, 2021; Kumar et al., 2020; Kumar et al., 2019).

In the IBRL, the critic (value) function’s parameters ϕ are updated with the following Bellman backup (Hu et al., 2024):

$$L\left(\phi \right)=E_{s_{t},a_{t},r_{t},s_{t+1}∼B}\left[{\left({\hat{Q}}_{\phi }\left(s_{t},a_{t}\right)-Q\right)}^{2}\right],$$ (1)

where

$$Q\leftarrow r_{t}+\gamma \underset{a^{\prime }\in \left\{a_{t+1}^{\text{IL}},a_{t+1}^{\text{RL}}\right\}}{\text{argmax}\;}Q_{\phi^{\prime }}\left(s_{t+1},a^{\prime }\right).$$ (2)

Here, ${\hat{Q}}_{\phi }\left(s_{t},a_{t}\right)$ is the estimated Q-value with states and actions sampled from the replay buffer B , while the target value $Q_{\phi^{\prime }}\left(s_{t+1},a^{\prime }\right)$ in Equation 2 is estimated using the current RL policy $a_{t+1}^{\text{RL}}$ or IL policy $a_{t+1}^{\text{IL}}$ . IBRL training starts with a replay buffer mixed with expert demonstrations and transitions collected during interactions, which introduces a mismatch between the current RL policy and behavior policy. Although IBRL allows for selecting actions from the IL policy, whose output is closer to the behavior policy in the demonstration, it relies on an accurate value estimation between $Q_{\phi^{\prime }}\left(s_{t+1},a_{t+1}^{\text{RL}}\right)$ and $Q_{\phi^{\prime }}\left(s_{t+1},a_{t+1}^{\text{IL}}\right)$ . However, because of the exploration noises during the online interaction, the future rollout states $s_{t+1}$ sampled from B are likely OOD relative to the offline demo buffer, D (Lee et al., 2022; Nakamoto et al., 2023; Zhou et al., 2024). When the IL policy proposes actions in these OOD states, the critic networks have no prior data for these state–action pairs and could assign a lower Q-value than the OOD actions proposed by the RL agent. As a result, the lower bounds brought by the IL policy fail if the RL policy is updated with bad OOD actions with an overestimated Q-value. Such errors could be corrected by attempting the OOD action during online interaction and observing its actual Q-value, but this, in turn, leads to insufficient policy exploration. Thus, finding a reliable and calibrated Q-value estimation is crucial for mitigating the bootstrapping error (Nakamoto et al., 2023).

Another disadvantage of bootstrapping error is that OOD actions selected by the RL policy during online interaction can lead to state distribution shift. When the IL agent fails to provide high-quality actions for the unseen interaction states, the exploration efficiency of IBRL will be degraded. Furthermore, although the IBRL has stated that both twin delayed DDPG (TD3) and soft actor–critic (SAC) can be used as RL policies for continuous control tasks (Hu et al., 2024), the authors exclusively used TD3 in their experiments due to its strong performance and high sample efficiency in challenging image-based RL settings. However, we argue that the deterministic RL algorithm, TD3, is less suitable for high-dimensional, continuous state-based tasks as it is more prone to overfitting offline data, converging to sub-optimal policies, and suffering from inefficient exploration (Haarnoja et al., 2018b). To prevent the RL policy from convergence to a sub-optimal policy because of overfitting, a maximum entropy stochastic RL method, SAC, is adopted.

## 3 Preliminaries

This section presents an overview of maximum entropy DRL and IBRL.

### 3.1 Maximum entropy deep reinforcement learning

For sample efficiency, off-policy DRL methods have been widely studied due to their ability to learn from past experiences. However, studies have also found that the off-policy DRL method struggles to maintain stability and convergence in high-dimensional continuous state–action spaces (Haarnoja et al., 2018b). To address this challenge, maximum entropy DRL has been proposed.

As the state–action spaces are continuous in the selected robotics tasks, we consider a Markov decision process (MDP) with continuous state–action spaces: an agent explores and interacts with an environment, at each time step t ; the agent observes the state $s_{t}$ , takes action $a_{t}$ based on the RL policy $\pi_{\theta }$ with parameters θ , and receives rewards $r_{t}$ . Different from standard RL, which aims to find a policy that maximizes the expected return in Equation 3

$$J\left(\pi \right)=\overset{T}{\underset{t=0}{\sum }}E_{\left(s_{t},a_{t}\right)∼\theta_{\pi }}\gamma^{t}r\left(s_{t},a_{t}\right),\;\gamma \in \left[0,1\right),$$ (3)

maximum entropy DRL aims to maximize the discounted reward and expected policy entropy $H\left(\pi \left(\cdot |s_{t}\right)\right)$ at each time step in Equation 4:

$$J\left(\pi \right)=\overset{T}{\underset{t=0}{\sum }}E_{\left(s_{t},a_{t}\right)∼\rho_{\pi }}\left[\gamma^{t}\left(r\left(s_{t},a_{t}\right)+\alpha H\left(\pi \left(\cdot |s_{t}\right)\right)\right)\right],$$ (4)

where T is the terminal time step, $\gamma \in \left.\left[0,1\right.\right)$ is the discount factor, and α is the temperature parameter, which determines the relative importance of the entropy term against the reward and thus controls the stochasticity of the optimal policy (Haarnoja et al., 2018b). With this objective, maximum entropy DRL methods have shown great potential in DRL-efficient online exploration with sparse reward settings (Hiraoka et al., 2021; Ball et al., 2023), consistent with the goal of this paper.

To apply maximum entropy RL in continuous spaces, one of the widely used methods, SAC (Haarnoja et al., 2018b), is applied.

### 3.2 Imitation bootstrapped reinforcement learning

IBRL is a sample-efficient DRL framework that combines a stand-alone IL policy with an off-policy DRL policy (Hu et al., 2024). First, IBRL requires an IL policy $\mu_{\psi }$ trained using expert demonstrations D . The goal of $\mu_{\psi }$ is to mimic an expert behavior and can be trained by minimizing a BC loss $L_{BC}$ in Equation 5:

$$L_{BC}\left(\psi_{\mu }\right)=E_{\left(s^{\prime },a^{\prime }\right)∼D}{\left\|\mu_{\psi }\left(s^{\prime }\right)-a^{\prime }\right\|}_{2}^{2}.$$ (5)

Then IBRL leverages trained $\mu_{\psi }$ to help the DRL policy $\pi_{\theta }$ with online exploration and its target value estimation, referred to as the actor proposal phase and the bootstrap proposal phase, respectively. In the actor proposal phase, IBRL selects between an IL action, $a^{IL}∼\mu_{\psi }\left(s_{t}\right)$ , and an RL action, $a^{RL}∼\pi_{\theta }\left(s_{t}\right)$ . The action with a higher Q-value computed by the target critic networks, $Q_{\phi^{\prime }}$ , is selected for the online interaction. Thus, the action selection module in IBRL is defined in Equation 6:

$$a^{*}=\underset{a\in \left\{a^{\text{IL}},a^{\text{RL}}\right\}}{\text{max}\;}Q_{\phi^{\prime }}\left(s,a\right).$$ (6)

Furthermore, to prevent local optimum Q-value update, the soft version of IBRL selects actions according to a Boltzmann distribution over Q-values instead of considering argmax .

Similarly, in the bootstrap proposal phase, the future rollout will be carried out by selecting the action by argmax or argsoftmax between $Q_{\phi^{\prime }}\left(s_{t+1},a_{t+1}^{\text{IL}}\right)\;\text{and}\;Q_{\phi^{\prime }}\left(s_{t+1},a_{t+1}^{\text{RL}}\right)$ . The critic networks $Q_{\phi }\left(s_{t},a_{t}\right)$ are updated as presented in Equation 1. The RL policy network, $a^{RL}∼\pi_{\theta }$ , is updated as in the selected off-policy DRL.

## 4 Methods

To reduce the exploration time wasted in correcting unreliable overestimated Q-values and, in turn, improve exploration efficiency, it is crucial for the policy to favor distributions with more stable Q-values. This motivates selecting batches with reliable Q-value evaluations when updating both the critic and policy networks. Previous studies have shown that the Q-value estimates of $Q_{\phi^{\prime }}\left(s_{t+1},a\left(s_{t+1}\right)\right)$ are only reliable when $\left(s_{t+1},a\left(s_{t+1}\right)\right)$ is sampled from the same distributions as the dataset used to train $\hat{Q}\left(s_{t},a_{t}\right)$ (Kumar et al., 2019; Nakamoto et al., 2023). In our critic network update process, instead of selecting between $Q_{\phi^{\prime }}\left(s_{t+1},\mu_{\psi }\left(s_{t+1}\right)\right)$ and $Q_{\phi^{\prime }}\left(s_{t+1},\pi_{\theta }\left(s_{t+1}\right)\right)$ , where both $\left(s_{t+1},\mu_{\psi }\left(s_{t+1}\right)\right)$ and $\left(s_{t+1},\pi_{\theta }\left(s_{t+1}\right)\right)$ could be OOD state–action pairs, we propose to select between $Q_{\phi^{\prime }}\left(s_{t+1},\pi_{\theta }\left(s_{t+1}\right)\right)$ and $Q_{\phi^{\prime }}\left(s_{t+1}^{\prime },\mu_{\psi }\left(s_{t+1}^{\prime }\right)\right)$ , where $s_{t+1}^{\prime }$ is only sampled from D . This modification ensures that $\left(s_{t+1}^{\prime },\mu_{\psi }\left(s_{t+1}^{\prime }\right)\right)$ is always from the same distribution as D , providing a reliable and calibrated Q-value estimates of the reference policy, whose values are on the similar scale as the true return value of D (Nakamoto et al., 2023). With D fixed, we compare the mean estimated return of $\left(s_{t+1},\pi_{\theta }\left(s_{t+1}\right)\right)$ sampled from B against the bootstrapping-error-free ground-truth mean return of D , thereby reducing the accumulated bootstrapping error in the action selection process. Thus, Equation 2 when updating the critic network becomes (Equation 7)

$$Q\left(s_{t},a_{t}\right)\leftarrow r_{t}+\gamma Q_{\phi^{\prime }}\left(s_{t+1},a^{*}\left(s_{t+1}\right)\right).$$ (7)

Compared with IBRL, the key modification is a simple action selection module, denoted as $a^{*}\left(s\right)$

$$a^{*}\left(s\right)=\left\{\begin{array}{cc} \mu_{\psi }\left(s\right),\; & {\bar{Q}}_{\phi^{\prime }}\left(s^{\prime },\mu_{\psi }\left(s^{\prime }\right)\right)>{\bar{Q}}_{\phi^{\prime }}\left(s,\pi_{\theta }\left(s\right)\right),\\ \pi_{\theta }\left(s\right),\; & \text{otherwise}, \end{array}\right.$$ (8)

where $\bar{Q}$ denotes the mean of estimated Q-values, s is the state from B , and $s^{\prime }$ is the state only sampled in D .

In the bootstrap proposal phase, the future rollouts $s_{t+1}$ are sampled randomly from B . One can select $s_{t+1}^{\prime }$ by finding the states closest to $s_{t+1}$ within D , to enable more precise comparisons between nearby state–action pairs. However, for implementation simplicity, current $s_{t+1}^{\prime }$ is uniformly random-sampled from D . By simple random sampling, the expected sample mean Q-value, ${\bar{Q}}_{\phi^{\prime }}\left(s^{\prime },\mu_{\psi }\left(s^{\prime }\right)\right)$ , from each batch converges to the population mean Q-value of the expert buffer (Rice, 2007). Therefore, even though the comparison is made across different states, it remains valid because we have compared the mean Q-values of the distributions induced by the IL policy and the RL policy.

Similarly, to align the policy strategy in the online interaction phase with the policy selected to propose future rollouts, the same action selection module (Equation 8) is used. With fewer OOD actions getting selected, the state distribution shift is also mitigated. However, if $\mu_{\psi }\left(s\right)$ fails to provide good or recovery actions toward any state distribution shift, the considered action selection module might fail as $Q_{\psi^{\prime }}\left(s^{\prime },\mu_{\psi }\left(s^{\prime }\right)\right)$ is not updated with fixed D , and the same bad behavior from the reference policy is repeatedly selected. Therefore, to leverage this action selection module for enhanced exploration efficiency, the initial online exploration states should lie within or near those in D , and the reference policy should remain robust under small shifts in the state distribution.

Furthermore, to prevent the RL policy from overfitting the demonstration dataset and converging on a sub-optimal policy, we propose to replace TD3 with SAC. In SAC, the critic parameter ϕ is updated by minimizing the soft Bellman residual:

$$J_{Q}\left(\phi \right)=E_{\left(s_{t},a_{t}\right)∼D}\left[\frac{1}{2}{\left(Q\left(s_{t},a_{t}\right)-{\hat{Q}}_{\phi }\left(s_{t},a_{t}\right)\right)}^{2}\right],$$ (9)

where $Q_{\phi }\left(s_{t},a_{t}\right)$ is estimated using Equation 10:

$$\begin{array}{cc} Q\left(s_{t},a_{t}\right) & \leftarrow r_{t}+\gamma \left(Q_{\phi^{\prime }}\left(s_{t+1},a^{*}\left(s_{t+1}\right)\right)-\right.\\ & \left.\alpha \log\pi_{\theta }\left(f_{\theta }\left(\epsilon_{t+1};s_{t+1}\right)|s_{t+1}\right)\right). \end{array}$$ (10)

The stochastic actor parameter θ is updated by minimizing the expected KL-divergence:

$$\begin{array}{cc} J_{\pi }\left(\theta \right) & =E_{s_{t}∼D,\;\epsilon_{t}∼N}\left[\alpha \log\pi_{\theta }\left(f_{\theta }\left(\epsilon_{t};s_{t}\right)|s_{t}\right)\right.\\ & \;\left.-Q_{\phi }\left(s_{t},\;f_{\theta }\left(\epsilon_{t};s_{t}\right)\right)\right], \end{array}$$ (11)

where the stochastic action is $f_{\theta }\left(\epsilon_{t};s_{t}\right)$ and $\epsilon_{t}$ is an input noise distribution, sampled from some fixed distribution (Haarnoja et al., 2018b). We propose that the distribution can be the demonstration D , but in this study, we only consider a simple Gaussian distribution N . $\log\pi_{\theta }\left(f_{\theta }\left(\epsilon_{t};s_{t}\right)|s_{t}\right)$ is the log-probability of the stochastic action $f_{\theta }\left(\epsilon_{t};s_{t}\right)$ under the current policy $\pi_{\theta }$ .

Finally, to leverage the robustness of the proposed framework toward the quality of the demonstration, we propose to choose offline DRL as the reference policy (IL policy in the IBRL framework) when the quality of the demonstration is unknown or imperfect. With strong sequential decision-making ability, offline DRL can be more robust to the demonstration quality than IL methods (Kumar et al., 2020; Fujimoto and Gu, 2021).

Combining all the modifications, DRLR is introduced in Algorithm 1; our new modifications are marked in red.

Algorithm 1 **DRLR.**

1: **Input:** Critic networks $Q_{\phi_{i}}\left(s,a\right)$ and target critic networks $Q_{\phi_{i}^{\prime }}$ with random initial parameter values; policy network $\pi_{\theta }$ and target policy network $\pi_{\theta^{\prime }}$ ;
2: Initialize replay buffer B and expert buffer D ;
3: Train an reference policy $\mu_{\psi }$ with expert buffer D by IL or offline RL.
4: **for** each episode M **do**
5:  Reset environment to initial state $s_{0}$ .
6:  **for** each time step t **do**
7:   Observe $s_{t}$ from the environment and compute IL action $a^{IL}∼\mu_{\psi }\left(s_{t}\right)$ and RL stochastic action $a^{RL}∼\pi_{\theta }\left(f_{\theta }\left(\epsilon_{t};s_{t}\right)|s_{t}\right)$

8:   Compute Q-values from the target critic networks $Q_{\phi_{i}^{\prime }}$ .
9:   Execute $a^{*}$ based on (equation 8).
10:   Store transition $\left(s_{t},a_{t},r_{t},s_{t+1}\right)$ in replay buffer B .
11:   Randomly sample a minibatch of N transitions from the replay buffer B and D , respectively.
12:   Update critic network parameters using Equation 9.
13:   Update actor network parameters using Equation 11.
14:   Update target networks.
15:  **end for**
16: **end for**

## 5 Experiment design and evaluation

In this section, experiments are designed and conducted in the simulation to evaluate the proposed method. The experimental design and evaluation aim to answer the following core questions.

### 5.1 How generalizable is DRLR across environments with varying reward densities and state–action space complexities?

To answer the question, the tasks selected in the problem statement are studied under both dense reward and sparse reward settings. For the bucket loading task, the state and action dimensions are 4 and 3, respectively. The details, such as reward design, domain randomization, and prior demonstration collection, are provided in Section 6. For the open drawer task, the state and action dimensions are 23 and 9, respectively. The details of the open drawer task are provided in Makoviychuk et al. (2021a). The original reward design for the open drawer task is dense and contains distance reward, open drawer reward, and some bonus reward for opening the drawer properly. To study the same task with a sparse reward setting, we simply set the distance reward gain to 0. To collect simulated demonstrations for the open drawer task, a TD3 policy was trained with dense, human-designed rewards. A total of 30 prior trajectories are recorded by evaluating the trained TD3 with random noise added to the policy output.

Both tasks are trained with Isaac Gym (Makoviychuk et al., 2021b). All experiments with the open drawer task were run with 10 parallel environments, using two different random seeds (10 and 11) to ensure robustness and reproducibility. All experiments with the bucket loading task were run in a single environment, using two different random seeds (10 and 11). The detailed configurations for training each task are shown in Section 8.1.

Question A is answered through the following evaluation results: Figure 5 demonstrates the performance of DRLR to learn the open drawer task with both sparse reward and dense reward, by achieving the highest reward in both reward settings; the results validate the robustness of DRLR toward varying reward densities. Figures 5, 6 present the performance of DRLR under different state–action space complexities. By outperforming IBRL on the open drawer task and achieving comparable reward in the bucket loading task, the results validate the ability of DRLR to generalize across varying levels of state–action space complexities.

### 5.2 How effective is the proposed action selection module in addressing the bootstrapping error and improving exploration efficiency during learning compared to IBRL?

**FIGURE 3:**
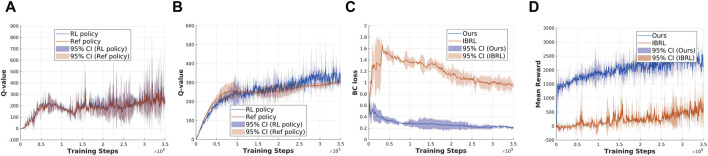
Exp2: Validation of the effectiveness of the proposed new action selection method using the open drawer task. **(a)** Q-value estimation with original IBRL. **(b)** Q-value estimation with our proposed action selection module. **(c)** BC loss. **(d)** Mean reward.

**FIGURE 4:**
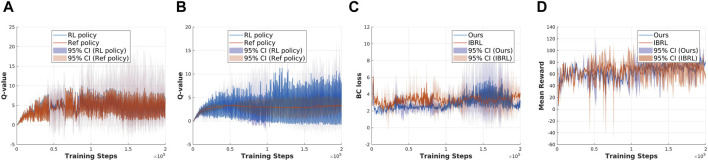
Exp3: Validation of the effectiveness of the proposed new action selection method using the bucket loading task. **(a)** Q-value estimation with original IBRL. **(b)** Q-value estimation with proposed new IBRL. **(c)** BC loss. **(d)** Mean reward.

To examine the effectiveness of the action selection module in addressing bootstrapping error and improving exploration efficiency, we conducted experiments in which only the action selection module of the original IBRL framework was replaced. The reference policy used is the IL policy, while the RL policy remains TD3 in both setups. Four criteria are recorded during training: 1) the Q-value of the Ref policy during action selection in the online interaction phase; 2) the Q-value of the RL policy during action selection in the online interaction phase; 3) BC loss: $L_{BC}\left(\pi_{\theta }\right)=E_{\left(s,a\right)∼B}{\left\|\pi_{\theta }\left(s\right)-a\right\|}_{2}^{2}$ , for measuring the difference between sampled actions in the replay buffer and the actions output by the RL policy; 4) reward convergence over training steps. Figures 3, 4 present a comparison of the considered criteria between the baseline IBRL and our proposed method across two selected tasks in the sparse reward setting.

The results for the open drawer task are shown in Figure 3. In Figure 3a, we compare the Q-value of the Ref policy and RL policy during action selection in the online interaction phase in the IBRL. The Q-values of the Ref policy appear closely estimated to that of the RL policy, and both Q-values have high variances during training. Combining the results of the BC loss between sampled actions and the agent’s output actions in Figure 3c indicates a mismatch between the updated policy and the behavior policy, suggesting that OOD actions are being selected due to the bootstrapping error discussed in Section 2. As a result, the Ref policy failed to get selected to provide reliable guidance, as reflected in the degraded performance in Figure 3d. Figure 3b presents a stable Q-value estimation of the Ref policy and a clear higher mean value compared with the RL policy in the early training steps, which aligns with the core idea of the IBRL framework. The corresponding BC loss in Figure 3c is significantly reduced by approximately 80% compared to the BC loss of IBRL, indicating that the bootstrapping error is effectively mitigated using our action selection method. Consequently, the Ref policy succeeded in efficient guidance throughout the RL training, as demonstrated by the improved reward convergence in Figure 3d. The proposed action selection module achieved a mean reward approximately four times higher than IBRL during the interaction steps.

The results for the bucket loading task are shown in Figure 4. Notably, the experiments of the bucket loading task were run in a single environment since it is computationally expensive to simulate thousands of particles in parallel environments. Thus, the results of the bucket loading tasks have higher variance than those of the open drawer task, where 10 environments are running in parallel. The results suggest that the action selection module has less effect on the low-dimensional state–action task, and the original IBRL can already score a near-optimal reward. This can also be attributed to the performance of the Ref policy. If the RL policy can easily acquire a higher Q-value than the Ref policy, the effect of our action selection module will be limited. Nevertheless, the stable Q-value estimation of the Ref policy in Figure 4b still validates the effectiveness of our action selection module in maintaining reliable Q-value estimations.

### 5.3 How effective is SAC in improving exploration efficiency during learning compared to the initial IBRL?

To examine the effectiveness of the SAC in improving exploration efficiency, we conducted 1) the original IBRL, denoted as ${\text{IBRL}}_{\mathrm{TD3}}$ ; 2) the IBRL with our action selection module, denoted as ${\text{Ours}}_{\mathrm{TD3}}$ ; 3) the IBRL with SAC to be the RL policy, denoted as ${\text{IBRL}}_{\mathrm{SAC}}$ ; 4) our DRLR framework, denoted as ${\text{Ours}}_{\mathrm{SAC}}$ . The Ref policy remains the IL policy in all setups.

**FIGURE 5:**
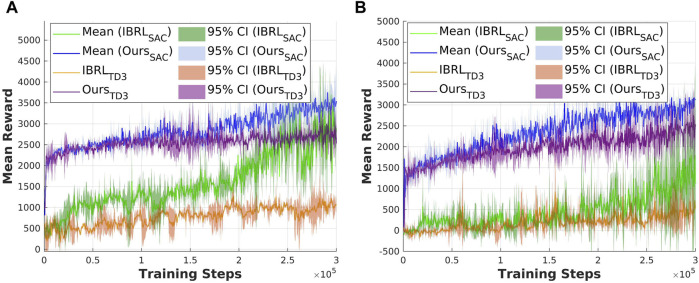
Exp4: Validation of the effectiveness of SAC using the open drawer task. **(a)** Dense reward setting. **(b)** Sparse reward setting.

**FIGURE 6:**
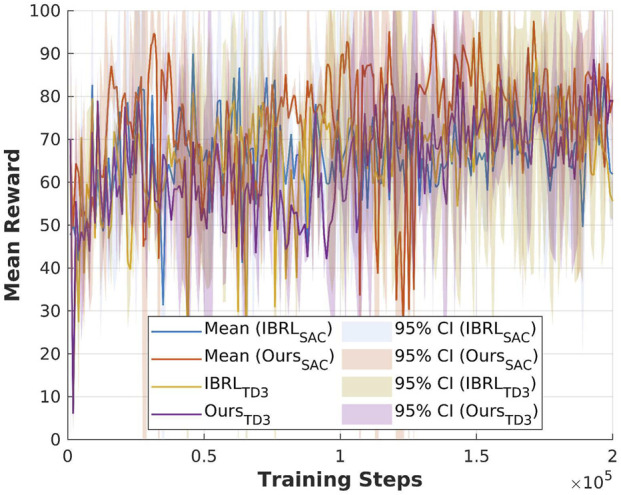
Exp5: Validation of the effectiveness of SAC using the bucket loading task.

The reward convergence over training steps is recorded as the main evaluation criteria. Figures 5, 6 present a comparison of the considered experiments across two selected tasks. The results for the open drawer task with varied reward settings are shown in Figure 5. The reward convergence suggests that with the same training steps, the experiments with SAC are able to explore higher rewards than those with TD3 , which converged on a sub-optimal reward. The results for the bucket loading task in sparse reward settings are shown in Figure 6. The results suggest that our method and IBRL achieve similar performance in low-dimensional state–action spaces.

The final evaluation results of each algorithm across two tasks are shown in Table 1. Table 1 shows that DRLR achieves the best evaluation performance in both tasks. In the open drawer task with sparse rewards, DRLR improves the averaged reward by approximately 347% , showing a dramatic improvement.

**TABLE 1:** Averaged rewards of evaluating each RL policy at the last time step over five episodes.

Task	${\text{IBRL}}_{\mathrm{TD3}}$	${\text{Ours}}_{\mathrm{TD3}}$	${\text{IBRL}}_{\mathrm{SAC}}$	${\text{Ours}}_{\mathrm{SAC}}$
Open drawer (dense)	1,055	2,735	2,747	3,455
Open drawer (sparse)	682.6	2,475	2,150	3,053
Bucket loading (sparse)	71.7	76.5	69.9	81.8

### 5.4 What is the impact of demonstration quality on the performance of our method?

**FIGURE 7:**
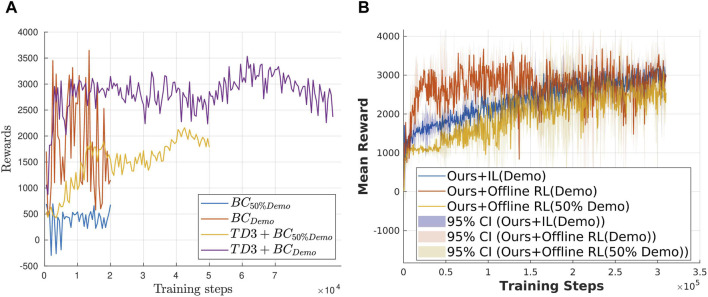
Exp6: Validation of the robustness of our framework toward varying demonstration qualities using the open drawer task. **(a)** Comparison between the IL policy and offline RL policy. **(b)** Reward convergence with the varying demonstration qualities.

To evaluate the robustness of the proposed method toward varying demonstration qualities, the following experiments were conducted: we fill the demonstration dataset with 1) 50% data from the random policy, denoted as 50%demo . 2) Sub-optimal demo: noise is added to the expert policy outputs. For simplicity, a BC policy is selected as the IL policy. A minimalist approach to offline RL, known as TD3 + BC (Fujimoto and Gu, 2021), is selected as our Ref policy. Due to the complexity involved in designing such experiments, only the open drawer task with sparse rewards, which is the most difficult to learn, is evaluated in the experiments. The results are shown in Figure 7. Figure 7a demonstrates that TD3 + BC can learn a good policy even from 50%demo , while BC cannot. Furthermore, TD3 + BC also learns a better policy using the sub-optimal demo. Figure 7b validates the robustness of our method toward varying demonstration qualities, by achieving the same level of rewards with both datasets.

To this end, we have demonstrated the effectiveness of the proposed method. The method is also applied to a real industrial application to showcase the implementation process and sim-to-real performance.

## 6 Real industrial applications

**FIGURE 8:**
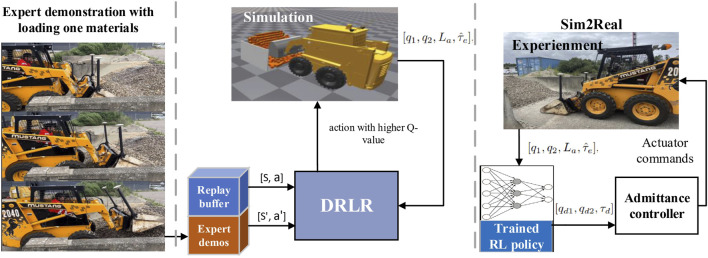
Illustration of the implementation of applying the proposed framework to the automatic wheel loader loading task.

This section presents an application of the proposed framework to the wheel loader loading task, where only a limited number of expert demonstrations are allowed to demonstrate the data efficiency. The detailed implementation is illustrated in Figure 8.

### 6.1 Bucket–media simulation

Before learning with the proposed framework, it is important to create an environment similar to the real world to enable policy exploration while applying domain randomization to deal with observation shifts. In the simulation, the wheel loader is configured with the same dynamic parameters obtained from a real machine. Because it is impractical to directly model the hydraulic actuation force or the bucket–media interaction force under different materials and geometries, this paper attempts to regularize the external torque rather than modeling it. We proposed to use admittance controllers to decrease the variances in the external torque by changing the position reference. The implementation of the admittance controller is provided in the Supplementary Appendices.

**FIGURE 9:**
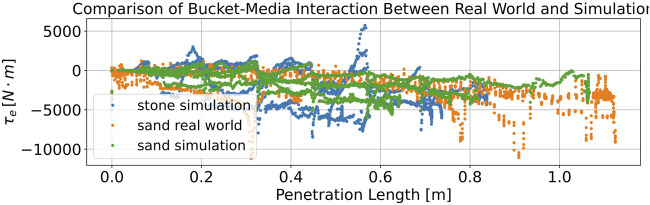
Comparison of the estimated external torque during penetration between simulation and real-world experiments. In the real-world experiment (orange), the external torque is measured while loading dry sand. In the simulation experiment, the external torque (green and blue) is generated by loading sand and stone piles, using the same penetration motion as in the real-world experiment.

Table 2 shows the parameters we randomized to simulate bucket–media interactions with different pile geometries and pile materials. A comparison of the estimated external torque during penetration of the pile between simulation and real-world experiments is presented in Figure 9. Different from real-world settings, the external torque is estimated from contact sensors in the simulation, due to the poor performance of the force sensor in Isaac Gym.

**TABLE 2:** Domain randomization parameters and their sampling ranges.

Domain randomization	Range
Density	$\left[1700\pm 100,2600\pm 100\right]kg/m^{3}$
Pile geometry	( 25° , 45° , 55° )
Particle friction	[0.3,0.4]
White noises on observations	[-1e^−4^, 1e^−4^]

### 6.2 DRLR implementation

Both the Ref and RL policies have four inputs, namely, $\left[q_{1},q_{2},L_{a},{\hat{\tau }}_{e}\right]$ , representing boom joint position, bucket joint position, advancing length, and estimated external torque, and three outputs, namely, $\left[q_{d1},q_{d2},\tau_{d}\right]$ , where $q_{d1},q_{d2}$ are desired position references for boom and bucket joint positions, respectively, and $\tau_{d}$ is the desired torque reference for admittance control that is only used during penetration.

**FIGURE 10:**
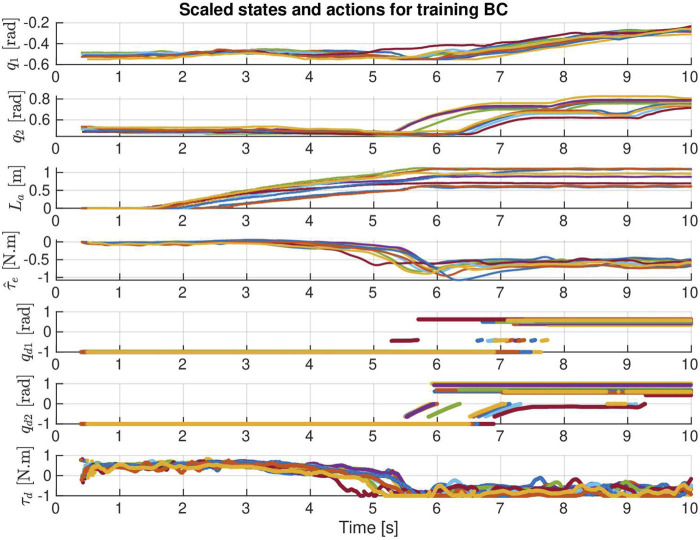
States–action pairs for training the Ref policy. Each curve represents the data recorded in one bucket loading demonstration.

To train the Ref policy, 10 expert demonstrations of loading dry sand piles with changing pile geometries are recorded. During demonstration, $\left[q_{1},q_{2},L_{a}\right]$ are directly used as inputs, and ${\hat{\tau }}_{e}$ is scaled at [−1,1] . Position references are acquired from the forward dynamics of the actuation signals sent in the demonstrations; they are first normalized and then used as $\left[q_{d1},q_{d2}\right]$ , while scaled ${\hat{\tau }}_{e}$ is directly assigned as $\tau_{d}$ . The state–action pairs that are used for training the reference policy are shown in Figure 10. For simplicity, BC is used to train the reference policy.

**FIGURE 11:**
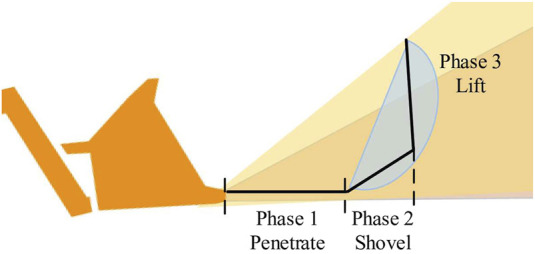
Three phases of the wheel loader loading process.

The wheel loader loading process can be divided into three phases, as shown in Figure 11: penetrate, shovel, and lift (Sarata et al., 2004).

To train DRL, the bucket loading task is divided into two sub-tasks as shown in Equation 12:

$$subtask=\left\{\begin{array}{cc} P_{1},\; & q_{d2}>-0.5,\\ P_{2}\&P_{3}\; & else \end{array}\right..$$ (12)

In phase 1, $P_{1}$ , the boom and bucket penetrate the pile with an admittance controller tracking $q_{d1},q_{d2},\tau_{d}$ , and the loader moves forward with a constant velocity. In phases 2 and 3, $P_{2}\&P_{3}$ , the controller switches to an inverse dynamics controller with only tracking the position references $q_{d1},q_{d2}$ , and the loader stops moving forward. The transition between $P_{1}$ and $P_{2}\&P_{3}$ is determined by the point at which the loader stops moving forward. Based on observing the demonstrations, this transition is identified when the desired bucket reference position $q_{d2}$ surpasses approximately −0.5.

The goal for the bucket loading task is to achieve a full bucket-fill rate and the boom–bucket joint reaching its designated end position, corresponding to the maximum allowable value within the position reference range. This leads to a natural sparse reward setting, where the reward only occurs at the end of the tasks. However, sparse reward requires a longer training time because it is more difficult for the RL agent to explore than dense reward settings. Although Shen and Sloth (2024a) demonstrated a successful performance with dense rewards, designing such rewards is challenging and may lead to sub-optimal actions. Since our framework has shown robust performance in sparse reward settings, a simpler sparse reward setting is designed in Equation 13:

$$r=\left\{\begin{array}{cc} R_{f}+R_{e},\; & T-50,\\ -10,\; & Fail,\\ 0,\; & Else, \end{array}\right.$$ (13)

where T represents the final step of an episode. A loading failure (Fail) occurs if the bucket-fill rate reward $R_{f}$ and the end reward $R_{e}$ do not achieve at least half of their maximum designed values by the end step T . The rewards $R_{f}$ and $R_{e}$ are defined as Equation 14:

$$R_{f}=\frac{V}{V_{\max}},\;R_{e}=1-\frac{d}{d_{\max}},$$ (14)

where $V_{\max}$ is the bucket capacity, V is the current bucket load volume, and $V={\hat{\tau }}_{e}/\rho_{\mathrm{rad}}gl_{1}$ , where $\rho_{\mathrm{rad}}$ is the particle density and $l_{1}$ is the length of boom. d is the Euclidean distance between the current boom–bucket joint position and the end position, while $d_{\max}$ is the Euclidean distance between the initial boom–bucket joint position and the end position.

### 6.3 Sim-to-real results

**FIGURE 12:**
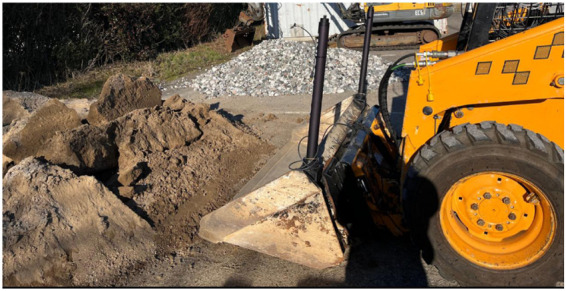
Experiment site showing MUSTANG 2040 operating in wet sand and stone pile fields.

The reward convergence results learning the bucket loading task are shown in Figure 6. The trained actor is deployed on a real machine MUSTANG 2040 operating in wet sand and stone pile fields. The experiment site is shown in Figure 12.

In the experiments, the inputs $\left[q_{1},q_{2}\right]$ are measured in radians with inertial measurement units (IMUs) mounted on the boom and bucket. $\left[L_{a}\right]$ denotes the forwarding distance of the loader, determined using GNSS antennas mounted on the machine. $\left[{\hat{\tau }}_{e}\right]$ is computed based on the pressure sensor readings obtained from both sides of the hydraulic pistons in the boom and bucket hydraulic pump. All the sensors operate at an update rate of 10Hz . The outputs $\left[q_{d1},q_{d2},\tau_{d}\right]$ are from the deployed Neural Networks (NNs), while the loader’s forwarding motion is manually controlled by an operator at a random speed. The operator halts the forward motion upon noticing the boom’s lift.

First, a two-sided admittance controller with both position and torque reference is tested. However, due to the high compaction nature of wet sand and stone pile, the downward curl of the bucket generates extremely large normal forces, causing the admittance controller to fail to track $\tau_{d}$ and, consequently, leading to boom and bucket vibrations during penetration and unstable outputs from the deployed actor network. These unstable NN outputs could result from a state distribution shift caused by the large normal forces during interaction with compacted material. In the simulation environment, such a compaction effect is not accurately modeled as the material pile is simulated using discrete particles that lack adhesive or cohesive properties. A penalty for causing such unsafe behavior should be considered in the future reward design.

Due to safety and stable performance, only a one-sided admittance controller is tested in the following experiments with position reference $\left[q_{d1},q_{d2}\right]$ and $\tau_{\mathrm{sat}}=800$ N.m to prevent the bucket from getting stuck.

**FIGURE 13:**
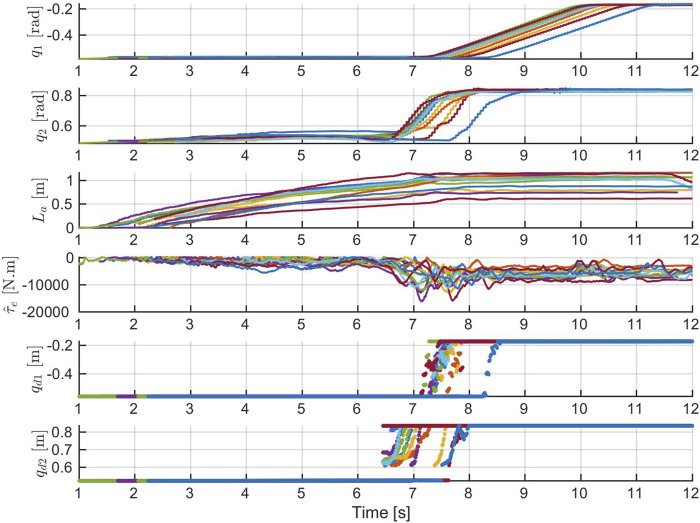
Sim-to-real results of 15 trials for loading stone with different pile geometries. Each curve represents the data recorded in one bucket-loading sim-to-real experiment.

To evaluate the policy, 25 experiments were carried out, involving 10 trials for loading wet sand and 15 trials for loading stone. Sim-to-real results for loading stones are presented in Figure 13. Despite changing environments, including pile geometries, material types, and forwarding velocities, all the experiments successfully loaded and lifted the materials. The average bucket-fill rates for loading sand and stone in the simulation and real-world experiments are provided in Table 3. To compare the sim-to-real performance in terms of the bucket-fill rate, the bucket-fill rates in simulation are also recorded and averaged over five episodes. The bucket-fill rate differences between simulation and real-world experiments may stem from environmental uncertainties present under real-world conditions, such as the irregular pile shapes.

**TABLE 3:** Average bucket-fill rates for simulation and real-world experiments.

Material	Simulation	Real world
Sand	93.71%	85.81%
Stone	90.01%	78.77%

## 7 Conclusion

This paper proposes and implements an exploration-efficient DRLR framework to reduce the need for extensive interaction when applying off-policy DRL to real-world robotic tasks. The designed experiments empirically validate the effectiveness of our framework in mitigating bootstrapping errors and addressing convergence to sub-optimal policies, ultimately reducing the exploration required to attain high-performing policies compared to IBRL. Furthermore, we demonstrated the implementation details for using the DRLR framework on a real industrial robotics task, wheel loader bucket loading. The sim-to-real results validate the successful deployment of the considered framework, demonstrating its potential for application to complex robotic tasks.

In future work, one could improve the action selection module by selecting $s_{t+1}^{\prime }$ by determining the states closest to $s_{t+1}$ within D using Euclidean or Mahalanobis distance, thereby facilitating more precise comparisons between neighboring state–action pairs. To better demonstrate the advantages of DRLR, it is necessary to compare it against established offline-to-online DRL baselines that explicitly addressed bootstrapping errors, such as CAL-QL, RLPD, and WSRL (Nakamoto et al., 2023; Ball et al., 2023; Zhou et al. 2024).

Moreover, one could also consider using deep ensembles to quantify the uncertainties in the demonstrations and utilize these uncertainties as prior data for the SAC entropy. Integrating the concepts of active learning and uncertainty-aware RL into the proposed framework could further improve the exploration efficiency.

## Data Availability

The raw data supporting the conclusions of this article will be made available by the authors, without undue reservation.
