# Supplementary material for: Solving robotics tasks with prior demonstration via exploration-efficient deep reinforcement learning
Source: Front Robot AI. 2026 Jan 12;12:1682200. doi: 10.3389/frobt.2025.1682200 (PMC12832430; doi:10.3389/frobt.2025.1682200)
Supplement: Supplementary file 1 [file Supplementaryfile1.pdf]

1 APPENDICES

1.1 Experiments configurations

Configuration	IBRL		DRLR	
	OpenDrawer	BucketLoad	OpenDrawer	BucketLoad
Learning rate	3e-4	3e-4	3e-4	3e-4
Batch size	128	128	128	128
Discount factor	0.99	0.99	0.99	0.99
Exploration noise Std.	0.1	0.1	–	–
Initial entropy	–	–	0.1	0.01
Learn entropy	–	–	True	False
Smooth noise Std.	0.1	0.1	–	–
Smooth noise clip	0.5	0.5	–	–
Dropout rate	0.1	0.1	–	–
Ensemble size of RED-Q	5	5	–	–
UTD	5	5	1	1
Replay buffer size	300k	200k	300k	200k

Table S1. Configuration of IBRL and DRLR across two tasks. The code for replicate experiments $1 \sim 7$ for DRLR and IBRL are available at `https://github.com/impala-shen/DRLR`. Our RL methods are developed using the RL library: skrl Serrano-Muñoz et al. (2023).

1.2 Additional comparisons between IBRL and DRLR.

To better understand the differences between IBRL and DRLR, additional comparisons including, 1) the Ref policy selection probabilities; 2) the bias of Q-return; 3) Mahalanobis Distance between sampled states to the expert states, are recorded for IBRL and DRLR. The additional comparisons are conducted on the new simulation platform: IsaacLab Mittal et al. (2025), using a task called $FrankaCabinet$. Since IsaacGym is now deprecated, all experiments were migrated accordingly. The task is executed with 128 parallel environments and trained over 5 random seeds (42–46). Expert demonstrations are generated using a trained PPO policy. Although the current performance on this task is still suboptimal, future work will be done to improve the results.

Figure S1 presents the Ref policy selection probabilities for DRLR and soft IBRL with temperature β $= 1$. As shown in Fig. S1a, DRLR selects the reference policy aggressively during the first 5×10^4 training steps, after which the selection probability gradually decrease to zero. This behavior aligns with the proposed action-selection module: DRLR leverages the reference policy early to obtain high-reward samples quickly, and once the RL policy becomes competent, it quickly takes over, eliminating the need to rely on the reference policy. In contrast, IBRL exhibits continuously increasing Ref policy selection throughout training, including near convergence. This trend indicates that IBRL remains dependent on the reference policies.

However, because of the dependence of IBRL on the Ref policy, there are cases where IBRL can obtain better reward convergence compared to DRLR. This occurs when the Ref policy is fairly strong to accomplish the task and when the bootstrapping error during training is small. To visualize these cases, 1) the Mahalanobis distance between sampled states and expert states, reflecting the state distribution shift, and 2) the bias of Q-return, are plot in Fig. S2 In Fig. S2 although DRLR exhibits a smaller Q-return bias (bottom plot), IBRL achieves better reward convergence (top plot) and a lower state distribution shift (middle plot). The lower state distribution shift in IBRL indicates that the optimized policy is close to the

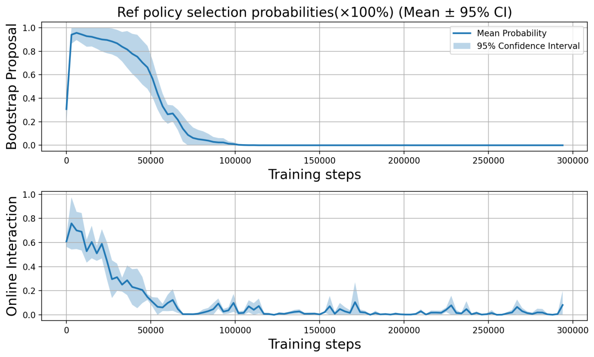

Figure S1a. Visualization of the Ref policy se-lection probabilities in DRLR.

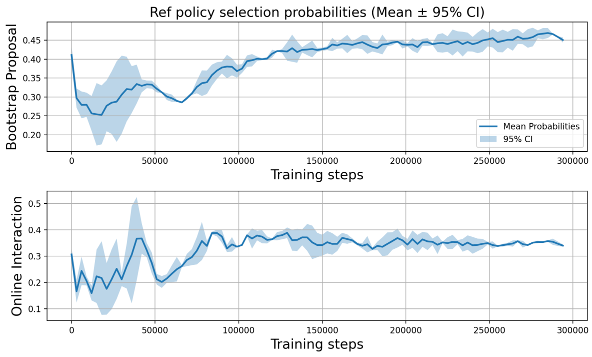

Figure S1b. Visualization of the Ref policy se-lection probabilities in IBRL.

Figure S1. Comparison of the Ref policy selection probabilities in DRLR and IBRL.

Ref policy. While in DRLR, the mean Q-estimation of the RL policy initially catches up quickly with that of the Ref policy due to the high Ref policy selection rate at the beginning. However, once the RL policy rapidly takes over the learning process, it struggles to explore state–action pairs that could yield higher rewards, and converging to sub-optimal performance. Addressing this limitation in DRLR requires more effective RL online exploration strategies and more precise comparisons between neighboring state–action pairs.

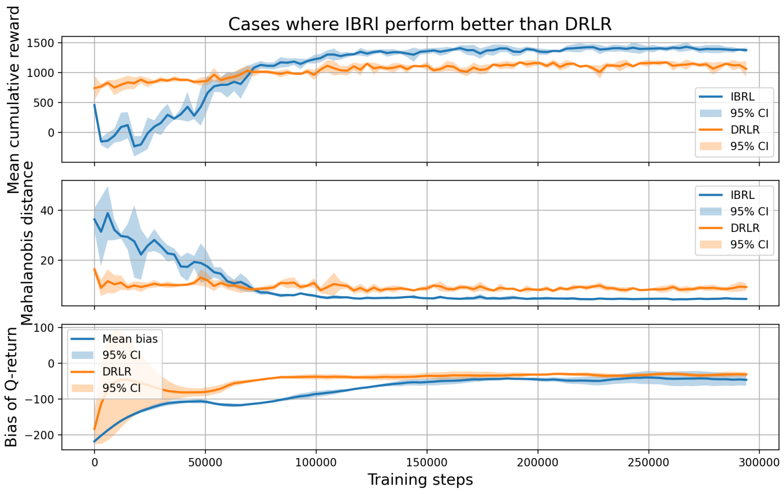

Figure S2. Cases where IBRL can obtain better reward convergence compared to DRLR because of its ability to obtain strong Ref policy.

1.3 Admittance controller

To control the wheel loader with an admittance controller, the wheel loader dynamics are modeled based on the Euler-Lagrange modeling:

$$M(q_i)\ddot{q}_i + n(q_i, \dot{q}_i) = \tau_i + \tau_e, \tag{1}$$

Where

$$n(q_i, \dot{q}_i) = C(q_i, \dot{q}_i)\dot{q}_i + \tau_f(\dot{q}_i) + g(q_i) \tag{2}$$

where $q_i, \dot{q}_i, \ddot{q}_i$ are position, velocity and acceleration of the joint, and the index $i = 1, 2$ is short for boom and bucket joint respectively. The non-linear effects, e.g. dead-zones caused by the hydraulic actuators are modeled as friction, τ_{f1} and τ_{f2} are torques caused by coulomb friction and viscous friction. τ_e is the external torque caused by interacting with the environment, it is estimated by a Sliding-mode Momentum Observer (MOB) proposed in Shen and Sloth (2024). The actuation torque τ_i can be obtained by the actuation force F_1, F_2 with the known hydraulic kinematics. F_i is obtained based on Yu et al. (2023):

$$F_i = p_{base}A_{base} - p_{rod}A_{rod} \tag{3}$$

where the p_{rod}, p_{base} are the pressure measurements from the pressure sensors installed on each side of the boom hydraulic cylinder. A_{rod}, A_{base} are the approximate areas of the rod and base side of the cylinder.

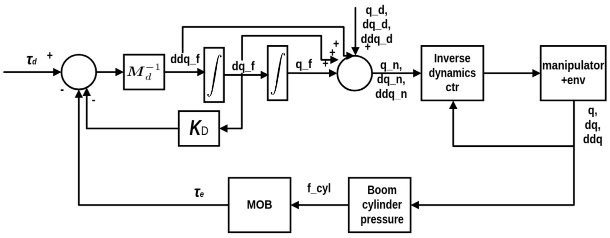

Figure S3. Proposed admittance controller.

The admittance controller starts from the measurements of torque difference, a mechanical admittance is used to motion variables from torque difference. The mechanical admittance law is given:

$$\tau_d - \tau_e = M_d\ddot{q}_f + K_{Dt}\dot{q}_f + K_P q_f. \tag{4}$$

1.3.1 Two-sided

According to Dobson et al. (2017), a two-sided admittance control has the best loading efficiency compared to a manual operator. A two-sided admittance controller is designed:

$$\ddot{q}_f = \begin{cases} -M_d^{-1}((\tau_{sat} - \tau_e) - K_D\dot{q}_f - K_P q_f), & \hat{\tau}_e > \tau_{sat} \\ M_d^{-1}((\tau_d - \tau_e) - K_D\dot{q}_f - K_P q_f), & else \end{cases} \tag{5}$$

where τ_{sat} is to prevent the bucket's downward curl from lifting the wheel loader or causing dramatically large normal force. τ_d is loading reference torque, which is output by RL.

1.3.2 One-sided

To prevent the bucket's downward curl from lifting the wheel loader or causing dramatically large normal force, a one-sided admittance controller is also designed:

$$\ddot{q}_f = \begin{cases} -M_d^{-1}((\tau_{sat} - \tau_e) - K_D\dot{q}_f - K_P q_f), & \hat{\tau}_e > \tau_{sat} \\ 0, & else \end{cases} \tag{6}$$

REFERENCES

Dobson, A. A., Marshall, J. A., and Larsson, J. (2017). Admittance control for robotic loading: Design and experiments with a 1-tonne loader and a 14-tonne load-haul-dump machine. *Journal of field robotics* 34, 123–150

Mittal, M., Roth, P., Tigue, J., Richard, A., Zhang, O., Du, P., et al. (2025). Isaac lab: A gpu-accelerated simulation framework for multi-modal robot learning. *arXiv preprint arXiv:2511.04831*

Serrano-Muñoz, A., Chrysostomou, D., Bøgh, S., and Arana-Arexolaleiba, N. (2023). skrl: Modular and flexible library for reinforcement learning. *Journal of Machine Learning Research* 24, 1–9

Shen, C. and Sloth, C. (2024). Safe operation for autonomous wheel loader using control barrier functions under unknown disturbances and input delay. In *2024 IEEE 20th International Conference on Automation Science and Engineering (CASE)* (IEEE), 4055–4061

Yu, S., Song, X., and Sun, Z. (2023). On-line prediction of resistant force during soil–tool interaction. *Journal of dynamic systems, measurement, and control* 145
